# Effect of Periodontitis on Dry Eye Disease Signs and Symptoms: A Cross-sectional Study

**DOI:** 10.3290/j.ohpd.b5573977

**Published:** 2024-07-19

**Authors:** Faruk Kaya, Basak Kiziltan Eliacik, Haci Koc, Mustafa Eliacik

**Affiliations:** a Associate Professor, Istanbul Medipol University, Department of Ophthalmology, Bagcılar, Istanbul, Turkey. Wrote the manuscript.; b Associate Professor, Department of Pedodontics, Hamidiye Faculty of Dentistry, Istanbul Health Sciences University, Istanbul, Turkey. Proofread the manuscript.; c Ophthalmologist, Private Dünya Eye Hospital, Eye Clinic, Sakarya, Turkey. Contributed substantially to discussion.; d Professor, Istanbul Medipol University, Department of Ophthalmology, Bagcılar, Istanbul, Turkey. Performed statistical evaluation.

**Keywords:** dry eye disease, inflammation, ocular surface, periodontitis, tear osmolarity

## Abstract

**Purpose:**

Gingivitis and periodontitis are oral disorders characterised by chronic inflammation, impacting the supportive structures around teeth due to bacterial accumulation. While the role of inflammation in both periodontitis and dry eye disease (DED) has been established individually, their potential association remains unclear. This study aimed to investigate the association between periodontitis and the manifestation of signs and symptoms related to DED in patients aged 18–40.

**Materials and Methods:**

A cross-sectional study was conducted involving healthy controls, DED patients with or without periodontitis, and patients with periodontitis without DED. Ophthalmic and oral examinations were performed, and demographic, ocular, and systemic disease data were collected. Statistical analysis was conducted using ANOVA and chi-squared tests.

**Results:**

A total of 684 participants were included in the study. Significant elevations in tear osmolarity levels, increased Ocular Surface Disease Index scores (OSDI), and decreased tear break-up time (TBUT) and Schirmer (ST-I) values were observed in DED patients with periodontitis compared to individuals with DED but without periodontitis, as well as control and periodontitis groups. Furthermore, higher neutrophil-to-lymphocyte ratios (NLR) were found in DED patients with periodontitis.

**Conclusion:**

The findings suggest an association between periodontitis and the severity of signs and symptoms related to DED. The study highlights the importance of interdisciplinary approaches in understanding the systemic implications of periodontal disease and its potential impact on ocular health.

Gingivitis and periodontitis are oral disorders marked by persistent and recurring inflammation.^28^ These conditions – based on the accumulation of bacteria in biofilms – primarily impact the supportive structures, including both the soft and hard tissues, that surround and hold the teeth in place. The occurrence of periodontal disease in the world’s population is as prevalent as caries, yet its systemic implications for general health have not been adequately recognised.^17^ Periodontitis is characterised as a localised infectious disease with the potential to induce a state of low-grade systemic inflammation, resulting in elevated levels of fibrinogen, C-reactive protein (CRP), and acute phase proteins in the bloodstream as a systemic reaction to inflammation.^3^ Interleukin-6 (IL-6), IL-1β, and tumor necrosis factor-α (TNF-α) are critical cytokines involved in regulating the production of the key acute phase protein CRP. Following tissue injury and infection, CRP level rapidly increase within hours, serving as evidence of a normal immune response by the host. Numerous studies have demonstrated a statistically significant correlation between CRP levels and the severity of periodontal disease.^1^^,^^6^^,^^24^^,^^27^ The study conducted by Eberhard et al^7^ demonstrated elevated levels of CRP and IL6 in the bloodstream of young, healthy volunteers diagnosed with gingivitis.

Dry eye disease (DED) is a pathological condition affecting the ocular surface, leading to ocular discomfort, compromised visual acuity, and instability of the tear film, which are recognised as hallmark symptoms of this disorder. Notable features of the disease include increased permeability of the tear film and inflammation of the ocular surface. While the exact pathogenesis of DED remains uncertain, it has been established that inflammation, whether localised or systemic, plays a significant role in the development and progression of the disease. The ocular surface of individuals with dry eye in DED has been found to exhibit elevated levels of IL-1, IL-6, IL-8, interferon-gamma, TNF-α, and matrix metalloproteinases, which are key inflammatory mediators.^19^ Özcan et al^23^ conducted a study that revealed elevated levels of inflammation markers, such as CRP, systemic immune-inflammation index (SII), neutrophil-to-lymphocyte ratio (NLR) and platelet-to-lymphocyte ratio (PLR), in the group of individuals with DED when compared to a control group consisting of healthy individuals.

Gingivitis and periodontitis, prevalent oral disorders characterised by enduring inflammation and bacterial accumulation, not only affect the oral cavity but also potentially induce systemic inflammation. This systemic inflammation, marked by elevated levels of inflammatory markers like CRP, is implicated in various diseases. Similarly, DED involves ocular surface inflammation and elevated inflammatory markers, including CRP. Both periodontal disease and DED share common inflammatory pathways, suggesting a potential interplay between oral and ocular health. Understanding this link could provide insights into the systemic implications of periodontal disease and its impact on ocular conditions like DED.

Based on this premise, we formulated the hypothesis that periodontitis, as a potential source of systemic inflammation, could contribute to the development and severity of signs and symptoms of dry eye. Due to findings indicating that cytokine levels, notably IL-6 and TNF-α, rise with age even in seemingly healthy individuals and in the absence of acute infection,^31^ our study focused on individuals aged 18 to 40. This age range was chosen to minimise potential confounding effects from age-related cytokine variations. Our objective was to explore the potential correlation between periodontitis and the expression of symptoms associated with DED within this specific age group.

## MATERIAL AND METHODS

The study was carried out from September 2020 to December 2022, adhering to the principles outlined in the Declaration of Helsinki. Ethical approval was obtained from the ethics committee of Istanbul Medipol University (Approval No: 10840098-772.02-E.58380). All participants involved in the study were provided with comprehensive information about the study’s purpose, procedures, and potential risks. Their voluntary participation was confirmed by obtaining written consent prior to the commencement of the study.

### Participants

This descriptive, cross-sectional study was conducted in a hospital setting and included various groups of participants: healthy controls, DED patients with or without periodontitis, and patients with periodontitis without DED. The patient selection process involved reviewing a database from Istanbul Medipol University Hospitals Complex, over the timespan from September 2020 to November 2022, and identifying individuals with diagnoses of “periodontitis,” “DED” and “periodontitis and DED.” The diagnostic codes used were H04.12 (DED) and K05.3 (chronic periodontitis). The collected data encompassed demographic information, ocular history, systemic diseases, ocular findings, and oral examinations. Ophthalmic examinations were also performed, and any patients from this cohort with pre-existing ocular diseases were excluded. Sample size calculation was conducted using the G*Power software program (Heinrich-Heine-Universität Düsseldorf, Düsseldorf, Germany).^8^ With a statistical power set at 0.9 and a type-I error rate of 5% for an ANOVA, a minimum sample size of 168 per group was determined. Accounting for a 10% potential dropout rate, a total of 186 patients were planned to be recruited for each group. To ensure comparability, 168 healthy control subjects matched in terms of gender, age, and body mass index were selected from patients attending our eye clinic for routine eye examinations.

The inclusion criteria encompassed individuals ages 18 to 40 years, diagnosed with DED according to the dry eye criteria established by the International Dry Eye Work Shop (DEWS) in 2007. These criteria include tear breakup time (TBUT) ≤ 10 s, Schirmer I test (ST-I) ≤ 10 mm/5 min, and positive conjunctival or corneal fluorescein staining (CS). Additionally, participants were required to have fully erupted, caries-free maxillary and mandibular incisors and first molars, absence of any systemic immune diseases or local ocular diseases, and a diagnosis of periodontitis based on the case definitions provided by the CDC-AAP working group, as documented in the reviewed patient files.

Participants were excluded if they had a history of smoking, current or recent drug use affecting the lacrimal functional unit, active ocular infections or allergies, ocular surface scarring, prior eye surgeries, or use of contact lenses. Additionally, exclusion criteria included primary Sjögren’s syndrome, other systemic rheumatic diseases, severe periodontal disease or therapy within the past 6 months, need for restorative and endodontic dental treatments, presence of orthodontic appliances, and clinical attachment loss. Participants with medical conditions affecting the neutrophil-to-lymphocyte ratio (NLR), such as diabetes mellitus, cardiovascular diseases, arterial hypertension, chronic obstructive pulmonary disease, malignancies, renal dysfunction, hepatic dysfunction, hematological or autoimmune disorders, were also excluded. Healthy control subjects underwent initial assessment by M.E. for routine check-up, excluding metabolic syndrome and systemic diseases, before referral to the ophthalmology department.

### Ophtalmologic Examinations

The ophthalmologists (F.K. and H.K.) were masked to the subjects’ oral health status during the ophthalmological examination. Patients initially completed the International Ocular Surface Disease Index (OSDI) survey.^11^ To assess participants’ daily screen time, an additional questionnaire was appended to the OSDI questionnaire.^29^ The questionnaire specifically addressed entertainment screen time, encompassing activities such as watching TV or videos, using a computer/tablet, playing video games, or utilising a cellphone for non-phone call purposes. Participants were asked to estimate their average duration of entertainment screen time per day. Subsequently, a comprehensive ophthalmological examination was conducted, including assessment of visual acuity, standardised anterior segment and fundus examination, anaesthetised Schirmer test (ST-I), TBUT, tear osmolarity measurement, and evaluation of corneal fluorescein staining (CS) as a part of the dry eye examination. All examinations were performed in a consistent environment with stable conditions (e.g., humidity, temperature). A minimum interval of 5 min was allowed between each test conducted on the ocular surface.

Tear osmolarity measurements were conducted using a TearLab osmometer (TearLab; San Diego, CA, USA). Tear samples were collected from the inferior lateral tear meniscus, and three consecutive measurements were taken. The mean value of these measurements was utilised for subsequent statistical analysis. The assessment of tear osmolarity emerges as a crucial tool in the assessment of ocular health, particularly in the context of DED. Supported by numerous studies,^12^^,^^26^^,^^36^ tear osmolarity testing not only provides objectivity and quantifiability but also remarkable specificity as a biomarker. Its accuracy exceeds that of traditional dry eye evaluation methods such as ST-I and CS. To assess CS, moistened fluorescein strips were introduced into the conjunctival sac, and the cornea was examined using a slit-lamp with a yellow barrier filter and cobalt blue illumination. The staining of the cornea was graded using the 6-point Oxford Scheme (ranging from 0 to 5).

**Fig 1:**
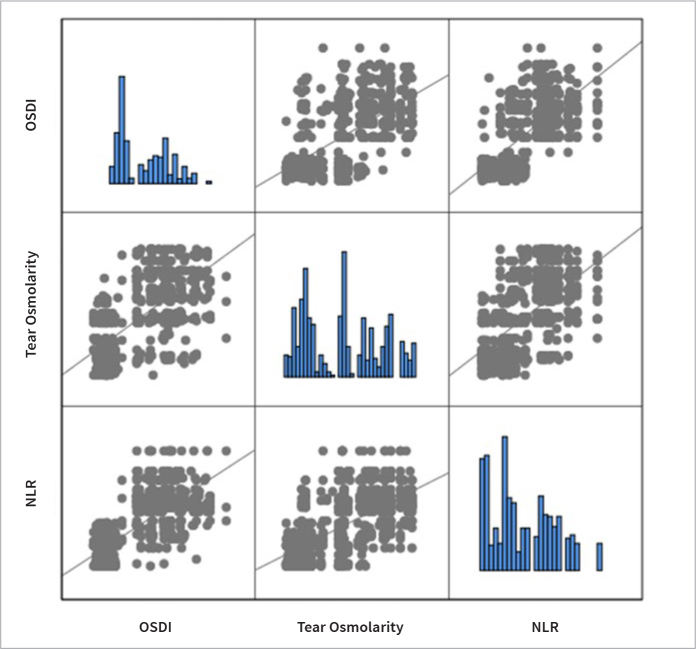
Correlation between neutrophil-to-lymphocyte ratio and tear osmolarity, and ocular surface disease index.

TBUT, defined as the time interval between the last complete blink and the appearance of the first dry spot, was assessed after instillation of 2% fluorescein staining under a cobalt blue filter, and the measurements were recorded (Fig 1). Three consecutive measurements were obtained, and their mean value was used for statistical analysis.

ST-I was performed with topical anaesthesia using a standardised filter strip (Bio-Tech Vision Care; Ahmedabad, India), and the amount of wetting was measured at the fifth minute.

Absolute neutrophil and lymphocyte counts were derived from the routine blood test data extracted from the hospital records of the patients, enabling the calculation of the NLR for each individual by dividing the neutrophil count by the lymphocyte count. NLR serves as a systemic inflammatory response marker, with inflammation triggering a rise in neutrophils and a decline in lymphocyte count, rendering their ratios a valuable tool for indirectly assessing both inflammatory status and cell-mediated immunity. To minimise potential confounding effects of investigations and treatments conducted throughout the hospital, the most recent and unaltered complete blood count values were considered, which were obtained closest to the patients’ ophthalmological tests and without any secondary medical interventions.

The statistical analysis was conducted utilising the SPSS 25.00 software package for Windows. Descriptive statistics were presented as mean values accompanied by their standard deviations (SD). The normality of distribution for each parameter was assessed using the Kolmogorov-Smirnov normality test. For the analysis of continuous variables such as OSDI, tear osmolarity, TBUT, ST-I, CS, and NLR, a one-way ANOVA was performed, followed by post-hoc Tukey’s tests for pairwise comparisons. Categorical variables were analysed using the chi-squared test. Statistical significance was set at p < 0.05 to discern differences between groups..

## RESULTS 

A total of 168 age-matched controls (group 1, 90 males and 78 females), 172 patients with concurrent DED and periodontitis (group 2, 92 males and 80 females), 175 DED patients without periodontitis (group 3, 89 males and 86 females), and 169 periodontitis patients (group 4, 86 males and 83 females) were included in this study. The mean age of the study subgroups was 29.1 ± 6.4, 29.0 ± 6.3, 28.4 ± 6.4, and 28.7 ± 6.3 years, respectively (p = 0.759). There were no statistically significant differences among the four groups in terms of sex, screen time, and ocular parameters, such as intraocular pressure and spherical equivalent (Table 1.

**Table 1:** Demographic and ocular characteristics of the study subjects

Variables	Controls (n = 168)	DED Patients with periodontitis (n = 172)	DED Patients without periodontitis (n = 175)	Patients with periodontitis without DED (n = 169)	p
Age	29.1 ± 6.4	29.0 ± 6.3	28.4 ± 6.4	28.7 ± 6.3	0.759*
Gender (M/F)	90/78	92/80	89/86	86/83	0.739†
Spherical equivalent (diopters, mean ± SD)	-0.138 ± 1.01	-0.127 ± 1.03	-0.151 ± 1,03	-0.116 ± 1.77	0.082*
Intraocular pressure (mm Hg)	16.3 ± 1.7	15.4 ± 1.6	16.57 ± 2.4‡	16.71 ± 2.9‡	0.728*
Screen time (minutes)	274,40 ± 161,18	293,89 ± 158,60	282,35 ± 161,98	286,75 ± 157,38	0.726*

* One way ANOVA, post-hoc Tukey’s test. †Chi-squared test. DED: dry eye disease.

**Fig 2:**
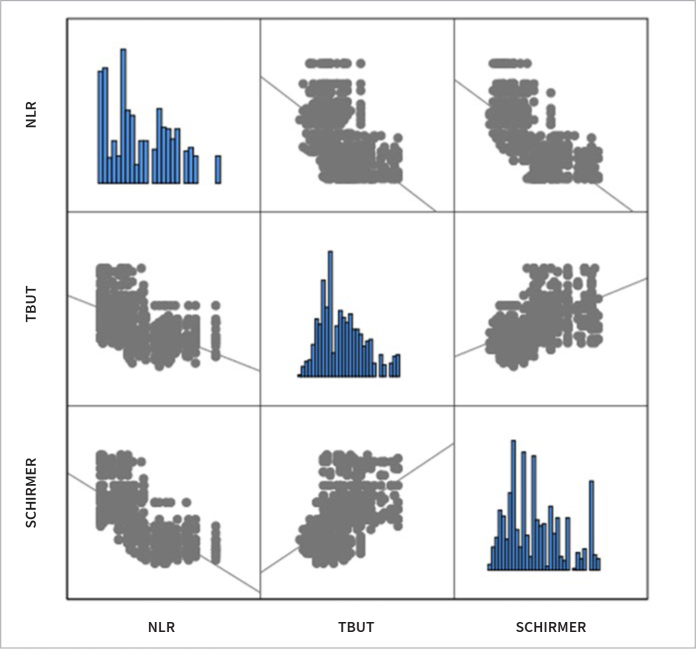
Correlation between neutrophil-to-lymphocyte ratio and tear break up time, and Shirmer I test.

The dry eye tests and mean NLR values of all groups are shown in Table 2. In the comparison between the group of patients with concurrent DED and periodontitis and all other groups, a statistically significant difference was observed in all dry eye tests, except for CS, as well as in the mean values of NLR (Tables 3–5). When comparing patients with periodontitis to healthy volunteers in the control group, a statistically significant difference was found solely in the mean values of tear osmolarity (p = 0.02). Upon examining the values of all participants in the study, a correlation analysis between NLR values and dry eye tests revealed a negative correlation between NLR and TBUT, ST-I, and CS, while a positive correlation was observed between NLR and tear osmolarity and OSDI values (Figs 1 and 2).

**Table 2:** Ocular tests and neutrophil-lymphocite rates among four groups

Groups		TBUT	OSDI	Tear osmolarity	Schirmer	Oxford	NLR
Healthy Volunteers	Mean	20.34	10.89	302.44	22.38	0.54	1.45
	Std. Deviation	5.53	3.60	9.54	6.87	0.56	0.32
DED	Mean	12.17	57.96	336.54	7.77	2.19	4.19
	Std. Deviation	3.52	19.53	14.24	3.78	0.77	0.76
DED with Periodontitis	Mean	10.11	54.18	332.26	9.93	2.36	3.89
	Std. Deviation	3.34	8.19	14.59	2.51	0.49	1.13
Periodontitis	Mean	19.77	13.28	306.36	21.62	0.64	2.20
	Std. Deviation	5.69	10.28	11.33	5.77	0.57	0.48

**Table 3:** Comparing tear break-up time and ocular surface disease index among four groups

Dependent variable	(I) groups	(J) groups	Mean difference (I-J)	Sig.	95% Confidence Interval	
					Lower bound	Upper bound
TBUT	1	2	8.17	0.00	6.87	9.48
		3	10.23	0.00	8.92	11.54
		4	0.57	0.68	-0.74	1.87
	2	1	-8.17	0.00	-9.48	-6.87
		3	2.06	0.00	0.75	3.37
		4	-7.61	0.00	-8.91	-6.30
	3	1	-10.23	0.00	-11.54	-8.92
		2	-2.06	0.00	-3.37	-0.75
		4	-9.67	0.00	-10.97	-8.36
	4	1	-0.57	0.68	-1.87	0.74
		2	7.61	0.00	6.30	8.91
		3	9.67	0.00	8.36	10.97
OSDI	1	2	-47.06	0.00	-50.41	-43.71
		3	-43.29	0.00	-46.63	-39.94
		4	-2.38	0.26	-5.73	0.97
	2	1	47.06	0.00	43.71	50.41
		3	3.77	0.02	0.43	7.12
		4	44.68	0.00	41.33	48.02
	3	1	43.29	0.00	39.94	46.63
		2	-3.77	0.02	-7.12	-0.43
		4	40.90	0.00	37.56	44.25
	4	1	2.38	0.26	-0.97	5.73
		2	-44.68	0.00	-48.02	-41.33
		3	-40.90	0.00	-44.25	-37.56

**Table 4:** Comparing tear osmolarity and Schirmer test 1 among four groups

Dependent variable	(I) groups	(J) groups	Mean difference (I-J)	Sig.	95% Confidence interval	
					Lower bound	Upper bound
Tear osmolairty	1	2	-34.10	0.00	-37.64	-30.56
		3	-29.82	0.00	-33.36	-26.28
		4	-3.92	0.02	-7.46	-0.38
	2	1	34.10	0.00	30.56	37.64
		3	4.28	0.01	0.74	7.82
		4	30.18	0.00	26.64	33.72
	3	1	29.82	0.00	26.28	33.36
		2	-4.28	0.01	-7.82	-0.74
		4	25.90	0.00	22.36	29.44
	4	1	3.92	0.02	0.38	7.46
		2	-30.18	0.00	-33.72	-26.64
		3	-25.90	0.00	-29.44	-22.36
Schirmer Test 1	1	2	14.61	0.00	13.20	16.03
		3	12.46	0.00	11.05	13.87
		4	0.76	0.51	-0.65	2.17
	2	1	-14.61	0.00	-16.03	-13.20
		3	-2.15	0.00	-3.57	-0.74
		4	-13.85	0.00	-15.26	-12.44
	3	1	-12.46	0.00	-13.87	-11.05
		2	2.15	0.00	0.74	3.57
		4	-11.70	0.00	-13.11	-10.28
	4	1	-0.76	0.51	-2.17	0.65
		2	13.85	0.00	12.44	15.26
		3	11.70	0.00	10.28	13.11

**Table 5:** Comparing corneal staining and neutrophil-to-lymphocyte ratio among four groups

Dependent variable	(I) groups	(J) groups	Mean difference (I-J)	Sig.	95% Confidence interval	
					Lower bound	Upper bound
Corneal staining	1.00	2.00	-1.65	0.00	-1.83	-1.48
		3.00	-1.82	0.00	-1.99	-1.65
		4.00	-0.10	0.48	-0.27	0.08
	2.00	1.00	1.65	0.00	1.48	1.83
		3.00	-0.17	0.06	-0.34	0.00
		4.00	1.56	0.00	1.39	1.73
	3.00	1.00	1.82	0.00	1.65	1.99
		2.00	0.17	0.06	0.00	0.34
		4.00	1.73	0.00	1.56	1.90
	4.00	1.00	0.10	0.48	-0.08	0.27
		2.00	-1.56	0.00	-1.73	-1.39
		3.00	-1.73	0.00	-1.90	-1.56
NLR	1.00	2.00	-2.74	0.00	-2.95	-2.53
		3.00	-2.44	0.00	-2.65	-2.23
		4.00	-0.75	0.00	-0.96	-0.54
	2.00	1.00	2.74	0.00	2.53	2.95
		3.00	0.30	0.00	0.09	0.51
		4.00	1.99	0.00	1.78	2.20
	3.00	1.00	2.44	0.00	2.23	2.65
		2.00	-0.30	0.00	-0.51	-0.09
		4.00	1.69	0.00	1.48	1.90
	4.00	1.00	0.75	0.00	0.54	0.96
		2.00	-1.99	0.00	-2.20	-1.78
		3.00	-1.69	0.00	-1.90	-1.48

The data that support the findings of this study are available from the corresponding author, F.K., upon reasonable request.

## DISCUSSION

In our study, we observed statistically significant elevations in tear osmolarity levels, increased scores on OSDI, and decreased TBUT and ST-I values in patients with DED and periodontitis compared to individuals with DED but without periodontitis, as well as the control and periodontitis groups. Additionally, higher NLR ratios were found in patients with DED and periodontitis.

Bacteremia commonly occurs in both general periodontal diseases and following dental examination or treatment, as well as during daily activities such as brushing, flossing, and chewing.^22^ These processes induce the release of proinflammatory cytokines from periodontal pockets in response to bacteremia. Gingival tissues that are chronically inflamed in patients with periodontitis have been found to exhibit elevated levels of cytokines, including IL-1, IL-6, IL-8, and TNF-α.^21^ Similarly, the gingival crevicular fluid of these patients contains high levels of these cytokines. The acute-phase reactant CRP is induced by interleukin 6 (IL-6) in Hep3B cells.^10^ Recent studies have reported increased blood levels of CRP in certain forms of inflammatory oral diseases, indicating an elevated acute-phase response triggered by infectious burdens and/or inflammation.^2^^,^^32^^,^^33^ CRP serves as a marker for assessing the individual’s inflammatory status. Cross-sectional studies have shown that patients with periodontitis have higher serum levels of CRP compared to healthy controls, which may explain the correlation between periodontal inflammation and dry eye disease.^5^^,^^18^^,^^25^ Interleukin-6 (IL-6) stimulates the liver to produce CRP and other acute-phase proteins.^35^ Inflammation has been shown to play a statistically significant role in the pathogenesis of non-Sjögren’s DED and Sjögren’s disease.^9^ In a long-term follow-up study by Lin et al,^16^ the association between exposure to periodontitis and primary Sjögren’s syndrome (pSS) was assessed, revealing a higher risk of pSS in patients with a history of periodontitis compared to those without a diagnosis of periodontitis. Another common finding is increased levels of autoantibodies and proinflammatory cytokines in DED patients. These inflammatory mediators can cause an immune invasion of the lacrimal gland, and disrupt its functions. It was reported that cytokines like IL-1b and TNFa could directly inhibite neurotransmitter release, which cause decreased lacrimal gland secretion.

The aetiological importance of tear film instability, hyperosmolarity, inflammation, and ocular surface damage have been well-documented in the cyclical disease process. It is evident that the presence of a “vicious circle” of inflammation plays a crucial role in the mechanism of DED, emphasising the need to interrupt this cycle in the treatment of the disease. Numerous studies have demonstrated that NLR, calculated as the absolute neutrophil count subtracted from the absolute lymphocyte count, is a simple yet informative investigation that provides valuable insights into the disease status of individuals with DED and other ocular diseases.^4^^,^^13^^-^^15^^,^^30^ These findings support the notion that inflammation likely plays an important role in the pathophysiology of DED and suggest that dry eye may be considered a systemic disease.^4^^,^^14^^,^^30^ Additionally, a study conducted by Özcan et al^23^ reported statistically significantly higher levels of inflammation markers, including CRP, NLR, and PLR, in the DED group compared to healthy controls.

Several studies indicate that inflammation is an important component of the pathophysiology of DED.^13^^,^^19^^,^^20^^,^^30^^,^^34^ To the best of our knowledge, this study represents the first report to highlight a potential association between periodontitis and DED.

Our study had several limitations that should be acknowledged. Firstly, the sample size was small, and the study was conducted at a single center, which may limit the generalisability of the findings. Additionally, the assessment of inflammatory markers or conjunctival redness, as well as meibography, were not included in the evaluation of the patients’ tear films, representing further limitations. Furthermore, we were unable to explore the correlation between the severity of periodontitis and dry eye clinical tests. It is evident that further research is warranted to elucidate the underlying mechanisms responsible for the relationship between oral conditions, CRP levels, and DED. Longitudinal investigations would be valuable in elucidating the temporal dynamics of this association, providing insights into the progression and potential causal mechanisms involved. Additionally, more comprehensive evaluations of inflammatory markers and clinical tests could deepen our understanding of the underlying pathophysiology and help identify biomarkers for early detection and targeted interventions. These approaches will contribute to advancing our knowledge and ultimately improving the management of both periodontitis and dry eye disease.

The current findings highlight three key outcomes that warrant consideration. Firstly, the role of periodontal disease in systemic inflammation should be approached with a broader perspective. It can be seen as either a consequence of an underlying hyperinflammatory process or as an integral part of systemic inflammation. The strong correlation between clinical manifestations of gingival inflammation and DED clinical tests underscores the relationship between local and systemic inflammatory processes. Additionally, thorough examination of the oral cavity in patients with DED may yield valuable clinical insights into disease activity. Therefore, integrating oral disease assessments with systemic inflammatory markers is crucial for comprehensively evaluating patients’ overall health status.

## CONCLUSION

In summary, our study demonstrated that patients diagnosed with both DED and periodontitis exhibited deteriorated tear film parameters when compared to individuals without periodontitis and the control group. It is noteworthy that patients with the coexistence of periodontitis and DED seemed to lack awareness of the potential association between their ocular symptoms and oral health. Based on these statistically significant findings, we strongly advocate for periodontologists and ophthalmologists, despite being from distinct disciplines, to comprehensively evaluate their patients for both ocular and oral symptoms. Furthermore, it is crucial to recognise the impact of periodontal health on overall ocular health in order to provide comprehensive care to these individuals.
